# Long-Term Outcomes after Convergent Procedure for Atrial Fibrillation

**DOI:** 10.3390/jcm13185508

**Published:** 2024-09-18

**Authors:** Borut Geršak, Veronika Podlogar, Tine Prolič Kalinšek, Matevž Jan

**Affiliations:** 1 Faculty of Medicine, University of Ljubljana, 1000 Ljubljana, Slovenia; bgersak@icloud.com; 2 Cardiovascular Surgery Department, University Medical Centre Ljubljana, 1000 Ljubljana, Slovenia

**Keywords:** atrial fibrillation, persistent atrial fibrillation, long-standing persistent atrial fibrillation, convergent procedure, hybrid ablation, catheter ablation

## Abstract

**Background**: The aim of this single-center retrospective study was to evaluate the long-term outcomes after the convergent procedure (CP) for treatment of AF. **Methods**: We analyzed the outcomes of patients that underwent CP from January 2009 until July 2020. A total of 119 patients with paroxysmal AF (23.5%), persistent AF (5.9%), or long-standing persistent AF (70.6%) that attended long-term follow-up were included. The outcomes were assessed 1 year after the CP and at long-term follow-up. At the 1-year follow-up, rhythm and AF burden were assessed for patients with an implantable loop recorder (61.2%). For others, rhythm was assessed by clinical presentation and 12-lead ECG. At long-term follow-up, patients with sinus rhythm (SR) or an unclear history were assessed with a 7-day Holter ECG monitor, and AF burden was determined. Long-term success was defined as freedom from AF/atrial flutter (AFL) with SR on a 12-lead ECG and AF/AFL burden < 1% on the 7-day Holter ECG. **Results**: At 1-year follow-up, 91.4% of patients had SR and 76.1% of patients had AF/AFL burden < 1%. At long-term follow-up (8.3 ± 2.8 years), 65.5% of patients had SR and 53.8% of patients had AF/AFL burden < 1% on the 7-day Holter ECG. Additional RFAs were performed in 32.8% of patients who had AF or AFL burden < 1%. At long-term follow-up, age, body mass index, and left atrial volume index were associated with an increased risk of AF recurrence. **Conclusions**: CP resulted in high long-term probability of SR maintenance. During long-term follow-up, additional RFAs were required to maintain SR in a substantial number of patients.

## 1. Introduction

Atrial fibrillation (AF) is a worldwide epidemic that results in significant burden on public health and healthcare systems [1,2]. The incidence and prevalence of AF have been consistently increasing throughout the years [2,3]. Patients with AF have an increased risk of heart failure, myocardial infarction, stroke, dementia, and mortality [4,5], and a higher AF burden decreases health-related quality of life [6,7,8].

Several treatments for AF have been developed with catheter ablation being a Class I indication for paroxysmal and persistent AF treatment when antiarrhythmic drug therapy fails [9]. Outcomes after catheter ablation have improved and are superior in comparison to pharmacological therapy alone, however, the success rates for persistent and long-standing persistent AF are still insufficient [10,11].

Convergent procedure (CP) is a multidisciplinary solution for the treatment of AF that involves epicardial ablation of the left atrium with a minimally invasive surgical approach and an endocardial catheter ablation. It was demonstrated to be superior in treating persistent AF compared to the endocardial-only ablation strategy in the CONVERGE trial [12,13]. Additionally, it was shown to be superior in improving the outcomes in patients with paroxysmal AF [14,15]. 

However, few studies were published exploring the long-term outcomes of the CP [12,16,17,18]. The objective of our retrospective analysis is to evaluate the very long-term outcome for patients with AF that underwent CP.

## 2. Methods

### 2.1. Patients

Our single-center retrospective analysis reports the long-term outcomes for patients that underwent CP from January 2009 to July 2020. During this period, 170 adult patients with symptomatic paroxysmal, persistent, or long-standing persistent AF, refractory to at least one antiarrhythmic drug Class Ic or III, received CP at the University Medical Centre in Ljubljana. Patients with paroxysmal AF included in this analysis were primarily included in a single-center randomized study comparing outcomes of CP versus catheter ablation [15]; no additional patients with paroxysmal AF were referred to CP after the completion of the study.

The CP protocol was reviewed and approved by the National Medical Ethics Committee.

### 2.2. Convergent Procedure

The procedure was performed under general anesthesia in a hybrid operating room equipped with fluoroscopy and electrophysiology mapping systems. Due to major safety concerns related to esophageal thermal injury during radiofrequency energy delivery, the initial procedural protocol was modified from February 2010 to include the following: 1. Use of a temperature probe for monitoring esophageal temperature rises during both the epicardial and endocardial procedure, with a cut-off of 38 °C. 2. Irrigation of ice-cold saline intrapericardially to cool the esophagus during radiofrequency energy delivery. 3. Postprocedural endoscopy of the esophagus for detection of thermal injury within 48 h after the procedure.

### 2.3. Epicardial Ablation

Access to the pericardial space was obtained through a laparoscopic transabdominal transdiaphragmatic approach or subxiphoid approach. After 2018, only a subxiphoid, transthoracic approach was utilized.

**Figure 1:**
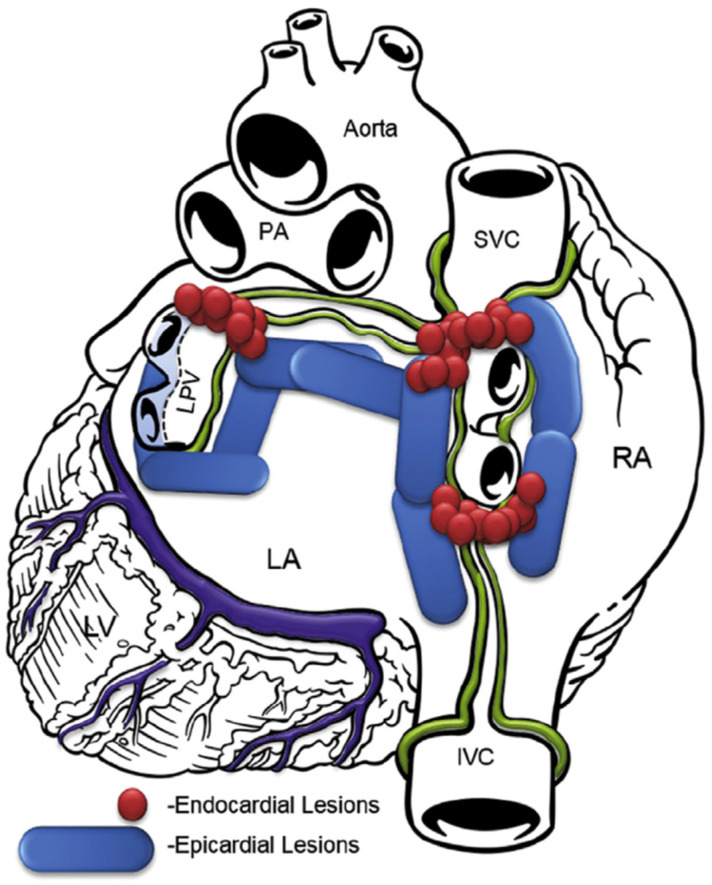
Lesion pattern before 2018. LA, left atrium; RA, right atrium; SVC, superior vena cava; IVC, inferior vena cava; PA, pulmonary artery; LPV, left pulmonary veins; LV, left ventricle.

In the transdiaphragmatic approach, access to the posterior wall of the left atrium was obtained under CO_2_ insufflation into the abdominal cavity using laparoscopic instruments inserted through three abdominal trocars, as described previously [19]. A pericardial window was created through the central tendon of the diaphragm and pericardium. The Subtle™ cannula (Atricure, Mason, OH, USA/nContact, Morrisville, NC, USA) was inserted into the oblique sinus, providing a direct view of the posterior left atrium through the endoscope. The epicardial radiofrequency (RF) ablation device Numeris^®^ or Episense™ (both Atricure, Mason, OH, USA/nContact, Morrisville, NC, USA) was introduced through the cannula, adjacent to the endoscope. Under direct endoscopic visualization, interconnected epicardial lesions were created, as shown in Figure 1. During epicardial ablation, ice-cold saline was injected into the pericardial sac to minimize heating of adjacent structures and periprocedural complications.

**Figure 2:**
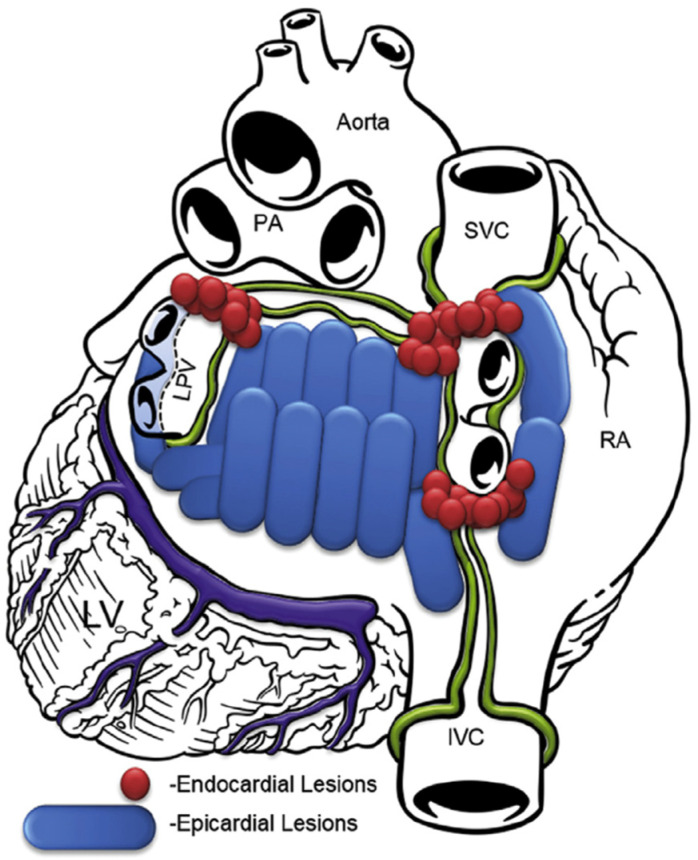
Lesion pattern since 2018. RA, right atrium; SVC, superior vena cava; IVC, inferior vena cava; PA, pulmonary artery; LPV, left pulmonary veins; LV, left ventricle.

In the subxiphoid approach, an incision was made directly under the sternum and the pericardial space was entered just above the diaphragm. The procedure was performed similarly to the transabdominal approach. Since 2018, the lesion set focused more on ablating the whole posterior left atrium, as shown in Figure 2. 

After epicardial ablation, incisions were closed, and endocardial ablation followed.

### 2.4. Percutaneous Endocardial Ablation

Endocardial access to the left atrium was obtained via femoral vein access and conventional transseptal puncture guided by fluoroscopy or intracardiac echocardiography (ICE). Mapping and ablation catheters were introduced into the left atrium. 

Since March 2013, NavX™ (Abbott, Abbott Park, IL, USA) was used to create electro anatomical LA maps to guide circumferential antral pulmonary vein isolation (PVI). Prior to March 2013, PVI was performed according to the ostial segmental isolation method.

While encircling the pulmonary veins’ ostia or antra, the breakthroughs between epicardial lesions along the pericardial reflections were identified. Those breakthroughs were ablated with Cool Flex™ or Flexability™ (Abbott, Abbott Park, IL, USA) to complete the PVI. Sites with no voltage were identified as necrotic tissue from previous epicardial ablation and were therefore not ablated. Entrance block by circular mapping catheter confirmed PVI.

### 2.5. Postoperative Management

If patients were not in sinus rhythm at the end of the CP, electrical cardioversion with direct current, biphasic, and using a maximal power setting of 360 joules was performed in the operating room. Other electrical or pharmacologic cardioversions and repeat catheter ablations during follow-up were directed by the referring physician. 

Initiation and discontinuation of antiarrhythmic drug therapy (AAD) and anticoagulation therapy throughout the follow-up were managed by the referring physician. Anticoagulation therapy was re-initiated after the CP for at least 3 months, long-term anticoagulation was instituted in patients with a CHA2DS2VASc score of 2 or more. 

After February 2010, all the patients received esophagoscopy before discharge [18].

### 2.6. Order of Procedures and Procedure Setting

The CP was, in general, performed as a first-time procedure without any prior invasive interventions. It was performed mainly at the same hospitalization as a single-setting procedure or rarely as a sequential epicardial first, endocardial later procedure on two different hospitalizations. When a single-setting approach was utilized, percutaneous endocardial ablation was performed immediately after the epicardial ablation was completed. In patients with arrhythmia recurrences, only endocardial catheter ablations were performed when needed, CP was never utilized for re-attempted procedures since there is a high probability of development of pericardial adhesions after the initial procedure.

### 2.7. Follow-Up Monitoring

The outcomes were assessed at one year after the procedure and at long-term follow-up by clinical presentation and rhythm monitoring. 

#### 2.7.1. One-Year Follow-Up

Most of the included patients were implanted with an implantable loop recorder (ILR [Reveal™ XT Insertable Cardiac Monitors; Medtronic, Minneapolis, MN, USA] or [Reveal LINQ™ Insertable Cardiac Monitors; Medtronic, Minneapolis, MN, USA]) within 24 h after CP. ILRs were implanted as a part of a study protocol for two previous studies from our group [15,18]. The rhythm and daily AF burden (AFB = % of time [day] in AF) were stored in ILR and then downloaded and assessed at the 1-year ambulatory visit. Any 30 s episodes of arrhythmia were assessed in patients with paroxysmal AF.

In patients that did not receive an ILR, rhythm at the 1-year follow-up was assessed by clinical presentation and with a 12-lead ECG recording.

#### 2.7.2. Long-Term Follow-Up

Long-term follow-up was performed between October 2021 and December 2022. Patients’ cardiology documentation was examined and patients were classified depending on the clinical presentation and 12-lead ECG recordings from previous ambulatory visits:

1. Patients with history of persistent or long-standing persistent AF who had sinus rhythm at previous ambulatory visits were assessed with a 7-day Holter ECG monitor. AF burden was determined;
2. A total of 8.4% (10/119) of patients with sinus rhythm on the 12-lead ECG recording at the last ambulatory visit refused the 7-day Holter ECG. For these patients, rhythm at long-term follow-up was assessed by clinical presentation and with a 12-lead ECG recording;
3. Patients with an unclear history of recurrent AF and known to have had persistent or long-standing persistent AF were telephoned and invited to an ambulatory visit. They were assessed with a 7-day Holter monitor. AF burden was determined;
4. Patients with history of persistent or long-standing persistent AF with known clinical history of recurrence of persistent AF during previous visits had a telephone call for demographic characteristics but were not evaluated with a 7-day Holter monitor;
5. Patients with paroxysmal AF were evaluated with a 7-day Holter monitor.

Class Ic and III AADs were assessed at 1-year follow-up and at long-term follow-up. Repeat catheter ablations prior to long-term follow-up were documented.

### 2.8. Definition of Procedural Endpoint and Outcomes

The procedural endpoint for the epicardial part of the CP was the visual overlapping of the ablation lesions on the posterior wall of the left atrium. The procedural endpoint for the endocardial part of the CP was the isolation of the pulmonary veins with the entrance block, confirmed with the circular mapping catheter. 

One-year success in the overall patient population and patients with persistent and long-standing persistent AF was defined as freedom from AF or AFL (atypical flutter) on the 12-lead ECG recording and AF/AFL burden < 1% on the ILR. For paroxysmal AF patients, every episode of AF or AFL lasting at least 6 min was considered a recurrence; this cut-off was chosen due to the intrinsic algorithm of the ILR used that stores single-lead ECGs of episodes lasting at least 6 min.

Long-term success was defined as freedom from AF or AFL with sinus rhythm on the 12-lead ECG recording and AF or AFL burden < 1% on the 7-day Holter ECG. For paroxysmal AF patients, every episode of AF or AFL lasting at least 30 s on the 7-day Holter ECG was considered a recurrence.

### 2.9. Complications

Major periprocedural complications were defined as events related to the procedure that happened in less than 30 days and prolonged hospital stay, resulted in rehospitalization, intervention, and/or had long-term negative impacts on patients’ health. 

Major long-term complications were defined as events related to the procedure that happened after the 30 days postprocedural period and resulted in rehospitalization, intervention, and/or had long-term negative impacts on patients’ health.

For the prevention of acute postprocedural pericarditis, all patients received an intrapericardial bolus of steroids at the end of the CP and NSAIDs orally were prescribed for at least two weeks.

### 2.10. Statistical Analysis

Normally distributed numeric measures are reported as the mean ± standard deviation (SD) and non-normally distributed variables as the median ± interquartile range. Categorical measures are presented as counts and percentages. 

Multivariate Cox Regression analysis with 95% confidence interval was performed to assess predictors of the outcomes. A *p*-value < 0.05 was considered statistically significant. For the statistical analysis, SPSS version 24.0 (IBM Corporation, Armonk, NY, USA) was used.

## 3. Results

### 3.1. Enrolment and Demographic Characteristics

One hundred and seventy (170) patients underwent endo- and epi-cardial CP from January 2009 to July 2020. A total of 51 were excluded; 38 were lost to follow-up and 13 were due to death before long-term follow-up. In 11 patients, death was not related to the ablation procedure. As reported previously, 2 out of the first 20 patients treated at our hospital died from atrio-esophageal fistula; after that, additional safety mechanisms were implemented in the procedure [18,19]. 

One hundred and nineteen (119) patients with endocardial and epicardial ablation and long-term-follow-up were included in the analysis. In total, 94.1% of included patients underwent CP between 2009 and 2017; 90.8% (108/119) of patients underwent CP in a single setting; and 9.2% (11/119) were treated in a staged CP, with the epicardial part of the procedure being performed before the endocardial one. Access to the pericardial space was obtained through a laparoscopic transabdominal transdiaphragmatic approach in 96.9% of patients (115/119) and through a subxiphoid approach in 3.4% of patients (4/119).

Demographic characteristics at the time of the CP and at long-term follow-up are shown in Table 1 and Table 2, respectively. Average age at the time of the CP was 58.4 ± 8.0 years and average age at the long-term follow-up was 66.7 ± 7.3 years. The majority of patients were male (77%). In total, 70.6% (84) of patients had long-standing persistent AF, 5.9% (7) had persistent AF, and 23.5% (28) had paroxysmal AF, as defined by the recommendations of the Heart Rhythm Society [20] and the European Society of Cardiology [9]. The average duration of AF before the CP was 4.9 ± 4.5 years.

At baseline, 40.3% of patients had a CHA2DS2VASc score ≥ 2; 14.3% had a history of heart failure; 65.5% experienced arterial hypertension; 6.7% had vascular disease (prior myocardial infarction, coronary artery disease, or peripheral artery disease); 1.7% had a prior stroke/TIA/thromboembolic event; and 9.2% had diabetes mellitus. At long-term follow-up, 72.3% of patients had CHA2DS2VASc a score ≥ 2; 37% had a history of heart failure; 76% experienced arterial hypertension; 17% had vascular disease; 2% had a prior stroke/TIA/thromboembolic event; and 13% had diabetes mellitus.

The median at the follow-up one year after the CP was 1.1 ± 0.3 years. The median at the long-term follow-up was 8.3 ± 2.8 years; the longest follow-up was 13.5 years after the CP.

### 3.2. Outcomes

#### 3.2.1. One-Year Follow-Up

**Figure 3:**
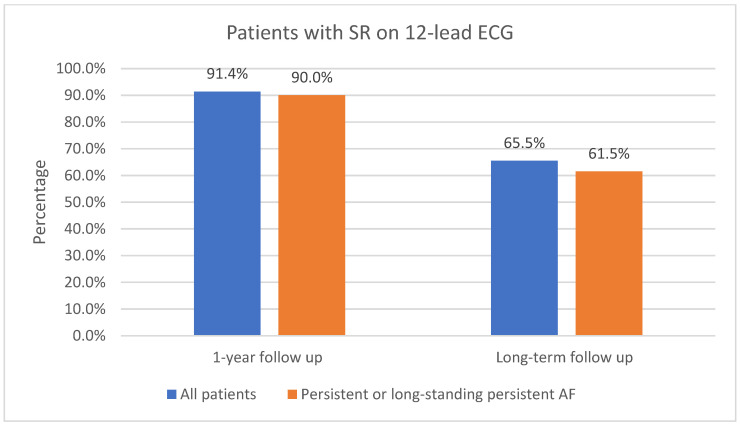
Patients in sinus rhythm on 12-lead ECG at 1-year follow-up and at long-term follow-up. SR, sinus rhythm; ECG, electrocardiogram, AF, atrial fibrillation.

**Figure 4:**
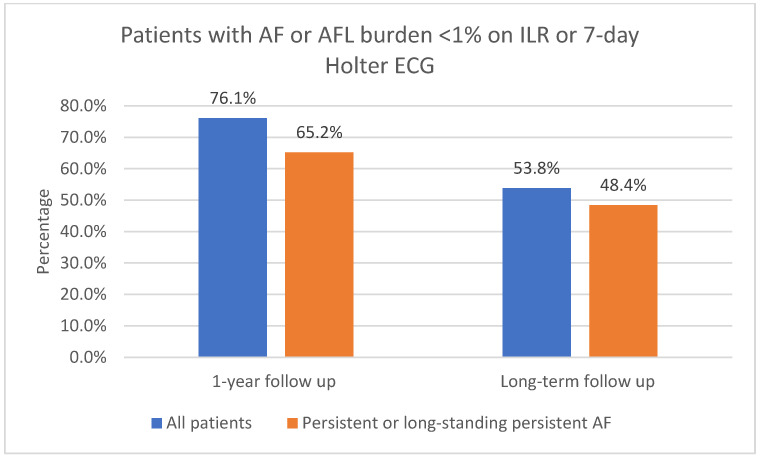
AF or AFL burden < 1% on ILR after 1-year follow-up and on 7-day Holter ECG after long-term follow-up. AF, atrial fibrillation; AFL, atrial flutter; ILR, implantable loop recorder; ECG, electrocardiogram.

The one-year follow-up data were available for 116 patients; 3 of the patients did not attend 1-year follow-up. As shown in Figure 3, 91.4% (106/116) of patients had sinus rhythm on the 12-lead ECG and 8.6% (10/116) had AF or AFL. In total, 73.6% (78/106) of patients in sinus rhythm were free off Class Ic or IIIAADs. A total of 61.2% (71/116) of patients had an ILR implanted. Of those patients who had an implanted ILR, 76.1% (54/71) had AF or AFL burden < 1%, as shown in Figure 4. Overall, 23.9% (17/71) had AF or AFL burden ≥ 1%, and 79.6% (43/54) of patients with AF or AFL burden < 1% were free off Class Ic or III AADs.

Ninety patients (90) with 1-year follow-up had persistent or long-standing persistent AF. As shown in Figure 3, 90.0% (81/90) of those patients had sinus rhythm on the 12-lead ECG and 10.0% (9/90) had AF or AFL. In total, 51.1% (46/90) of patients with persistent or long-standing persistent AF had an implanted ILR; of those, 65.2% (30/46) had AF or AFL burden < 1%, as shown in Figure 4. Overall, 34.8% (16/46) had AF or AFL burden ≥ 1%.

Twenty-six (26) patients with the 1-year follow-up had paroxysmal AF. An ILR was implanted in 23 patients; of them, 69.5% (16/23) had no episodes of AF or AFL lasting 6 min or more on the ILR.

No additional catheter ablations were performed in the first year.

#### 3.2.2. Long-Term Follow-Up

Long-term follow-up data were available for all of the included patients (119). As shown in Figure 3, 65.5% (78/119) of patients had sinus rhythm on the 12-lead ECG and 34.5% (41/119) had AF or AFL. In total, 85.9% (67/78) of patients in sinus rhythm on the 12-lead ECG were free off Class Ic or III AADs. Additional catheter ablations were performed in 44.5% (53/119) of all patients, including additional catheter ablations in 34.6% (27/78) of patients who had sinus rhythm on the 12-lead ECG.

Of all the included patients, 53.8% (64/119) had AF or AFL burden < 1%, as shown in Figure 4. In total, 51.3% (61/119) had no episodes of AF/AFL/AT lasting 30 s or more on the 7-day Holter ECG. Overall, 87.5% (56/64) of patients with AF or AFL burden < 1% on the 7-day Holter ECG were free off Class Ic or III AADs. Additional catheter ablations were performed in 32.8% (21/64) of patients who had AF or AFL burden < 1% on the 7-day Holter ECG.

Long-term follow-up data were available for 91 patients with persistent or long-standing persistent AF. Of all the included patients with persistent or long-standing persistent AF, 61.5% (56/91) had sinus rhythm in the 12-lead ECG, as shown in Figure 3; 48.4% (44/91) had AF or AFL burden < 1%, as shown in Figure 4; 86.4% (38/44) of them were free off Class Ic or III AADs. In total, 45.1% (41/91) had no episodes of AF or AFL lasting 30 s or more on the 7-day Holter ECG. Additional catheter ablations were performed in 38.6% (17/44) of these patients.

Long-term follow-up data were available for 28 patients with paroxysmal AF.

Of all the included patients with paroxysmal AF, 71.4% (20/28) had no episodes of AF or AFL lasting 30 s or more on the 7-day Holter ECG.

### 3.3. Predictors of Recurrence

Multivariate Cox Regression analysis did not identify any predictors significantly associated with increased risk of AF recurrence after one year.

At long-term follow-up, the multivariate Cox Regression analysis identified three predictors associated with increased risk of AF recurrence: age (HR 1.15, 95% CI 1.02–1.29, *p* = 0.023), body mass index (BMI) (HR 1.31, 95% CI 1.05–1.63, *p* = 0.018), and left atrial volume index (LAVI) (HR 1.08, 95% CI 1.01–1.15, *p* = 0.030). Other predictors were not significantly associated with AF recurrence.

### 3.4. Complications

Only complications in patients that completed the long-term follow-up are reported and shown in Table 3. A total periprocedural major complication rate of 10.1% was observed. Three patients developed cardiac tamponade (2.5%). Three patients had hemorrhage (2.5%): one from the abdominal wall that resulted in exploratory laparotomy and a blood transfusion with 4 units; one was bleeding intraabdominally due to abdominal adhesions from a previous cholecystectomy, which required surgical evacuation of hematoma and transfusion of 2 units of blood; and one hematemesis related to a previously unknown gastric ulcer. One patient with bilateral pneumonia required rehospitalization (0.84%). One patient with a pseudoaneurysm of the superficial femoral artery was treated surgically (0.84%). Right phrenic nerve paresis was observed in one patient (0.84%) and ulnar nerve compression (0.84%) in one patient. One patient developed sepsis one day after the procedure, which resolved with appropriate therapy (0.84%). One patient had acute decompensation of chronic cor pulmonale that resolved with therapy (0.84%). 

Three patients had possible mucosal thermal injury to the anterior wall of the esophagus that resolved after therapy with protein pump inhibitors. No other esophageal injuries were detected.

No patients experienced stroke during follow-up.

The major long-term adverse events rate was 2.5% as three ventral hernias required surgery later.

## 4. Discussion

Our retrospective study showed that the CP in our mixed group of patients with AF resulted in high probability of sinus rhythm maintenance of 76.1% at one year and 53.8% at long-term follow-up after 8.3 years; a majority of those patients (79.6% at one year and 87.5% at long-term follow-up) were off AADs. Additionally, in a subgroup of patients with persistent or long-standing persistent AF, we found a high probability of sinus rhythm maintenance of 65.2% at one year and 48.4% at long-term follow-up. We found that age, BMI, and LAVI were predictors of long-term outcomes. AF recurrence importantly increased with time. 

The multicenter, randomized convergent trial demonstrated the superiority of CP in patients with long-standing persistent AF with 73.7% effectiveness regardless of AAD use compared to 44.4% in the catheter ablation arm at 12 months [21]. A recently published randomized clinical trial, HARTCAP-AF, showed 89% and 36% freedom from any supraventricular tachyarrhythmia lasting >30 s off AADs at 12 months after convergent ablation and catheter ablation, respectively [22]. Similarly, Varzaly et al. performed a meta-analysis where sinus rhythm maintenance at 79.4% was demonstrated after CP at 19 months follow-up [23].

Importantly, outcomes for sinus rhythm maintenance after CP have been shown to decline over years. In a recent meta-analysis, Eranki et al. showed freedom from AF of 78.2% and 73.6% at 1 and 3 years after CP, respectively [16]. In the latest study, Pannone et al. reported arrhythmia-free survival without AADs in non-paroxysmal AF 76.7% at 12 months, 67.5% at 24 months, 60.5% at 36 months, 53.6% at 48 months, and 46.1% at 60 months [17].

Long-term results that are available for catheter ablation demonstrate a low success rate in catheter ablation of persistent and long-standing persistent AF. In the Hamburg study, a success rate of 45% was reported after multiple ablation procedures at ≥50 months [24]. In a meta-analysis, Ganesan et al. reported 3-year freedom from AF in patients with non-paroxysmal AF at 41.6% and 77.8% following a single-catheter ablation and multiple procedures, respectively [25]; however, most of the studies included in the meta-analysis performed follow-up with 24-h Holter, transtelephonic ECGs and some only performed symptom-related follow-up, which are methods of low sensitivity for detection of AF recurrence. The analysis of the CIRCA-DOSE trial showed that sensitivity of detecting AF recurrence increased with the duration of rhythm monitoring. Serial short-duration Holter ECG monitoring (24-/48-h) missed a substantial number of recurrences and less accurately estimated AF burden; however, serial long-term monitors (14-day) had sensitivity closer to the gold standard of continuous ECG monitoring [26]. 

Our results for short-term and mid-term outcomes are consistent with studies involving CP mentioned earlier and previous studies from our group [15,18,27]. Importantly, our current study reports the longest follow-up after CP, with a 53.8% freedom from AF/AFL recurrence at a median of 8.3 years. However, to reach the high probability of sinus rhythm maintenance at long-term follow-up, 32.8% of patients required at least one additional catheter ablation.

It is suggested that non-pulmonary vein substrate, located mainly on the posterior wall of the left atrium, as well as left atrial remodeling significantly contributes to sustaining arrhythmia in persistent and long-standing persistent AF [28,29]. More extensive and durable transmural lesions of the posterior wall with direct ablation of connections between endo- and epicardium and autonomic ganglia located in the epicardial fat pads [30]—resulting in homogenization of the posterior wall [31]—could play a major role in maintaining sinus rhythm after CP.

### 4.1. Complications

We report a rate of 10.1% for periprocedural major complications and a rate of 2.5% for major long-term adverse events. The complication rate is high; however, our cohort included patients from the beginning of the implementation of the CPs in our center. As previously reported, within the first twenty patients who underwent the CP, two patients developed atrio-esophageal fistulas resulting in death. An extensive hospital investigation resulted in the implementation of additional safety measures such as esophageal temperature monitoring, termination of RF energy dictated by the esophageal temperature increase, cooling of the pericardial space during epicardial ablation, minimization of endocardial ablation along the posterior wall of the left atrium, and implementation of postprocedural esophagoscopy [19]. We believe that these modifications by our group, implemented since February 2010, resulted in widespread use of CP on a global level because they clearly demonstrated safety from atrio-esophageal fistulas if the CP was performed in a single setting. Since then, no atrio-esophageal fistulas or deaths occurred. Our results are consistent with previous research, which shows major complication rates after CP between 0 and 20.8%, with a pooled complication rate of 5.53% [16,32,33,34]. In a single-center study, Larson et al. showed that transitioning from a transdiaphragmatic to a subxiphoid approach enhances the safety profile and significantly reduces complications. They reported a major complication rate of 23% and 3.8% after CP with the transdiaphragmatic or subxiphoid approach, respectively. Most complications were related to entering the abdominal cavity [30]; however, the subxiphoid approach results in poorer accessibility to the anterior segments of pulmonary veins, hence, only the posterior left atrial wall can be ablated epicardially, which could potentially reduce procedural success. 

Importantly, the complication rate of CP is clearly higher compared to the catheter ablation; this was shown in our previous study including patients with paroxysmal AF where we reported a rate of periprocedural major complications in the CP group of 12.5% versus 0% in the catheter ablation group [15]. Consequently, caution and strict patient selection is advised when considering CP for the treatment of paroxysmal AF.

### 4.2. Limitations

Our study has some obvious limitations. Firstly, it is a single-center retrospective study, which somewhat reduces the importance of our conclusions in comparison to some prospective and randomized studies referenced in our manuscript. Secondly, there was some possible bias related to the inclusion of patients as a substantial number of patients that had received the CP in our center refused to perform a long-term ambulatory visit. Thirdly, our study included a relatively small sample size, which can lead to an overestimation of the effect size. The data for the analysis of the predictors were incomplete and led to a smaller sample in the final analysis of the predictors. Fourthly, there was poor uniformity of follow-up since ILRs were used for most patients during short-term follow-up and 7-day Holter recordings were used at long-term follow-up. Clearly, the information obtained from both rhythm monitoring methods is only partially comparable, especially regarding the arrhythmia burden assessment. On the other hand, our group clearly demonstrates the evolution of CP from its initial phase to a fully developed invasive procedure, which was eventually implemented in major centers around the world.

## 5. Conclusions

In the longest reported follow-up to date, the CP for treatment of AF was shown to result in a high long-term probability of sinus rhythm maintenance. Additional catheter ablations were required to maintain sinus rhythm in a substantial number of patients with persistent and long-standing persistent AF.

## Figures and Tables

**Table 1:** Baseline demographic characteristics at CP. AF, atrial fibrillation; PLAX LA, parasternal long axis left atrial dimension; LAVI, left atrial volume index; LVEF, left ventricular ejection fraction; CP, convergent procedure; TIA, transitory ischemic attack, CHA2DS2VASC–C, congestive heart failure; H, arterial hypertension; A2, age ≥ 75 years; D, diabetes mellitus; S2, stroke/TIA/thromboembolism; V, vascular disease; A, age ≥ 65 years; SC, sex category.

Demographic Characteristics	Baseline at CP
Male, N (%)	92 (77)
Age, years (SD)	58.4 (8.0)
Body mass index, kg/m^2^ (SD)	29.6 (4.9)
AF duration, years (SD)	4.9 (4.1)
AF type, N (%)	
Persistent	7 (5.9)
Long-standing persistent	84 (70.6)
Paroxysmal	28 (23.5)
PLAX LA, cm (SD)	4.5 (0.6)
LAVI, mL/m^2^ (SD)	47 (13)
LVEF, % (SD)	55 (11)
Type of CP, N	
CP—single setting	108 (90.8)
CP—staged	11 (9.2)
Chronic heart failure, N (%)	17 (14.3)
Heart failure with reduced ejection fraction, N (%)	4 (3.4)
Arterial hypertension history, N (%)	78 (65.5)
Vascular disease, N (%)	8 (6.7)
Stroke/TIA/thromboembolic event, N (%)	2 (1.7)
Diabetes mellitus type II, N (%)	11 (9.2)
CHA2DS2VASc, N (%)	
CHA2DS2VASC 0	25 (21.0)
CHA2DS2VASC 1	46 (38.7)
CHA2DS2VASC 2	30 (25.2)
CHA2DS2VASC 3	10 (8.4)
CHA2DS2VASC 4	7 (5.9)
CHA2DS2VASC 5	1 (0.8)
CHA2DS2VASC 6	0 (0.0)
CHA2DS2VASC 7	0 (0.0)

**Table 2:** Demographic characteristics at long-term follow-up. TIA, transitory ischemic attack, CHA2DS2VASC–C, congestive heart failure; H, arterial hypertension; A2, age ≥ 75 years; D, diabetes mellitus; S2, stroke/TIA/thromboembolism; V, vascular disease; A, age ≥ 65 years; SC, sex category.

Demographic Characteristics	At Long-Term Follow-Up
Chronic heart failure, N (%)	44 (37.0)
Arterial hypertension history, N (%)	90 (75.6)
Vascular disease, N (%)	20 (16.8)
Stroke/TIA/thromboembolic event, N (%)	2 (1.7)
Diabetes mellitus, N (%)	16 (13.4)
CHA2DS2VASc, N (%)	
CHA2DS2VASC 0	10 (8.4)
CHA2DS2VASC 1	23 (19.3)
CHA2DS2VASC 2	26 (21.8)
CHA2DS2VASC 3	29 (24.4)
CHA2DS2VASC 4	17 (14.3)
CHA2DS2VASC 5	9 (7.6)
CHA2DS2VASC 6	4 (3.4)
CHA2DS2VASC 7	0 (0.0)
CHA2DS2VASC 8	1 (0.8)
Antiarrhythmic drugs	
Class Ic, N (%)	4 (3.4)
Class III, N (%)	13 (10.9)
Beta-blockers, N (%)	59 (49.6)
Class IV, N (%)	9 (7.6)

**Table 3:** Periprocedural major complications.

Complications	
Tamponade, N (%)	3 (2.5)
Stroke, N (%)	0
Esophageal ulcer/perforation/fistula, N (%)	0
Bleeding, N (%)	3 (2.5)
Infection, N (%)	2 (1.68)
Vascular complication, N (%)	1 (0.84)
Phrenic nerve palsy, N (%)	1 (0.84)
Peripheral nerve compression, N (%)	1 (0.84)
Acute worsening of previous heart disease, N (%)	1 (0.84)
Pulmonary vein stenosis (clinical screening), N (%)	0
Overall, N (%)	12 (10.1)

## Data Availability

The data underlying this article will be shared on reasonable request to the authors.
